# The Harris Lectures

**Published:** 1867-06

**Authors:** 


*The Harris Lectures.*—From the *Twenty-Eighth Annual Circular* of the Baltimore College of Dental Surgery, for the Session of 1867-68, just issued, it will be seen that the Faculty of this College have inaugurated a series of Lectures, to which they have given the name of their late distinguished President, and which they are confident will prove an important adjunct to the regular course, and very interesting to the Student.

The series will consist of twenty or thirty lectures, delivered on certain evenings throughout the session.

The subject of each lecture will be some point in Dental Surgery, or some specialty in Dental Mechanism, which the lecturer has made a particular subject of study.

Arrangements for the ensuing session are not yet completed, but a number of names of gentlemen are given, who have already kindly consented to assist in the work of Dental Education.

From the high character and position in the profession of the Lecturers, we anticipate an interesting and instructive series, and feel confident that this movement will contribute greatly to the benefit of both the student and the institution.

The Faculty propose in future circulars to give the names of the Lecturers, and the time, and subject of each lecture of the series.

